# Virological activity in treatment-naïve HBeAg-negative HBV-infected adult patients: A community-based study in Jiangsu, China

**DOI:** 10.1097/MD.0000000000021179

**Published:** 2020-07-17

**Authors:** Xiangjun Zhai, Liguo Zhu, Jie Jiang, Ci Song, Hong Peng, Jiao Qian, MingHao Zhou, Yan Zhou, Qungang Wang, Jianfang Xu, Zhijian Wang, Hongjian Liu, Min Fan, Zhibin Hu, Hongbin Shen, Fengcai Zhu

**Affiliations:** a Jiangsu Province Center for Disease Prevention and Control, Nanjing; b NHC Key Laboratory of Enteric Pathogenic Microbiology, Jiangsu Provincial Center for Disease Control and Prevention; c Key Laboratory of Infectious Diseases, School of Public Health, Nanjing Medical University; d Jiangsu Key Lab of Cancer Biomarkers, Prevention and Treatment, Jiangsu Collaborative Innovation Center for Cancer Personalized Medicine; e Department of Epidemiology, School of Public Health, Nanjing Medical University, Nanjing; f Zhangjiagang Center for Disease Prevention and Control, Zhangjiagang; g Danyang Center for Disease Prevention and Control, Danyang; h Taixing Center for Disease Prevention and Control, Taixing, People's Republic of China.

**Keywords:** chronic hepatitis B, community-based investigation, HBeAg-negative hepatitis B, liver function

## Abstract

Nowadays most of the hepatitis B virus (HBV) infected population are adults, among which hepatitis B e antigen (HBeAg) negative infection occupied the largest proportion of HBV infection in China. HBeAg-negative patients are heterogeneous, and the corresponding interventions are different. Therefore, it is worth researching the infection characteristics of HBeAg-negative patients to help guide the interventions.

A total of 11,738 treatment-naïve HBeAg-negative adult patients were randomly selected, and their demographic and medical history information were collected. The liver biochemistry, and HBV infection biomarkers including hepatitis B surface antigen (HBsAg), hepatitis B surface antibody (anti-HBs), HBeAg, hepatitis B e antibody (anti-HBe), hepatitis B core antibody (anti-HBc), and hepatitis B virus deoxyribonucleic acid (HBV-DNA) levels were tested. The infection characteristics and their influencing factors were explored.

Sixty percent of the patients presented HBV-DNA-positive, of which 31.2% had HBV-DNA level higher than 2000 IU/mL, and 16.5% had HBV-DNA level higher than 20,000 IU/mL. HBV-DNA levels tended to increase along with the increasing of age, and the male patients had significant higher HBV-DNA levels than the female patients. Twenty-four percent of the patients had abnormal transaminase. The male patients were more vulnerable to abnormal transaminase (30.0%) than the female patients (18.4%). Fifty-five percent patients with HBV-DNA ≥20,000 IU/mL presented abnormal alanine aminotransferase (ALT) or aspartate transaminase (AST), which was significantly higher than that of patients with HBV-DNA levels below 20,000 IU/mL (19.0–21.7%). Multivariate logistic regression analyses revealed that the male patients and the patients with higher viral load had higher risk of having abnormal liver function.

A considerable number of HBeAg-negative patients were virological active and had liver damage. It is necessary and urgent to carry out regular active interventions for the chronic HBV-infected patients.

## 1 Introduction

Chronic hepatitis B virus (HBV) was widespread in China before hepatitis B vaccine was used. The prevalence of hepatitis B surface antigen (HBsAg) was about 10% across all age groups.^[1,2]^ China introduced hepatitis B vaccination program in children in 1992, and three national representative serologic surveys conducted in 2002, 2006, and 2014 showed that the HBsAg prevalence among vaccinated populations decreased steadily and significantly.^[3–5]^ HBsAg prevalence among age groups 1 to 4 years, 5 to 14 years, and 15 to 29 years in 2014 were 0.3%, 0.9%, and 4.4%, respectively. HBsAg prevalence among 1 to 29 years has declined from 10.1% to 2.6% during 1992 to 2014.^[5]^ But in Chinese adult populations who were not involved in the hepatitis B immunization program, the HBsAg prevalence did not change much with the prevalence still higher than 8.0%. Given that most of the 9.3 million chronic HBV-infected people in China are adults,^[4]^ the HBV infection is not fully under control.

Chronic HBV infection has a wide clinical spectrum, ranging from asymptomatic carrier status to cirrhosis and hepatocellular carcinoma (HCC). In general, the course of chronic HBV infection can be classified into four different phases, That is, the “immune tolerant phase,” the “immune clearance phase,” the “immune control phase,” and the “immune escape phase.”^[6]^ These phases are characterized by different patterns of hepatitis B e antigen (HBeAg) status, HBV viral load and serum transaminase concentration. Briefly, “Immune tolerant phase” is characterized by the presence of serum HBeAg, very high levels of hepatitis B virus deoxyribonucleic acid (HBV-DNA) and alanine aminotransferase (ALT) persistently within the normal range according to traditional cut-off values. “Immune clearance phase” is characterized by the presence of serum HBeAg, high levels of HBV-DNA and elevated ALT. “Immune control phase” is characterized by the presence of serum hepatitis B e antibody (anti-HBe), undetectable or low (<2000 IU/mL) HBV-DNA levels and normal ALT according to traditional cut-off values. “Immune escape phase” is characterized by the lack of serum HBeAg usually with detectable anti-HBe, and persistent or fluctuating moderate to high levels of serum HBV-DNA (often lower than in HBeAg-positive patients), as well as fluctuating or persistently elevated ALT values.

Certain courses in HBV-infected patients are different and related to the prognosis of the disease. High HBV-DNA levels have been proved to be one of the risk factors of cirrhosis and HCC, especially in HBeAg-negative patients.^[7]^

In areas with high prevalence of HBV such as China, most individuals are infected perinatally, by vertical transmission or in early childhood.^[8]^ The majority of chronic HBV infection acquired before childhood will present with immune-tolerant status initially, experience the immune clearance phase later and finally enter the inactive phase after HBeAg seroconversion accompanied by cessation of HBV replication and remission of liver disease.^[6]^ HBeAg-negative patients are not homogenous. Some may be constant viral-inactive and have sustained remission, but others may be viral-reactive and experience disease progression including development of cirrhosis and HCC.^[9,10]^ Although most of the existing chronic hepatitis B infection in Chinese adults is composed of HBeAg-negative infection pattern, the data about viral reactivity in these patients based on community population are scarce. With the Hepatitis B comprehensive management demonstration project (HBCMDP) conducted in Jiangsu province, China, a large community-based HBsAg screening was performed in adults and a large number of chronic HBV-infected patients were diagnosed. The demographic, serological, virological, and liver functional characteristics of HBeAg-negative hepatitis B infected patients were reported as follows.

## 2 Methods

### 2.1 Patients selection

**Figure 1:**
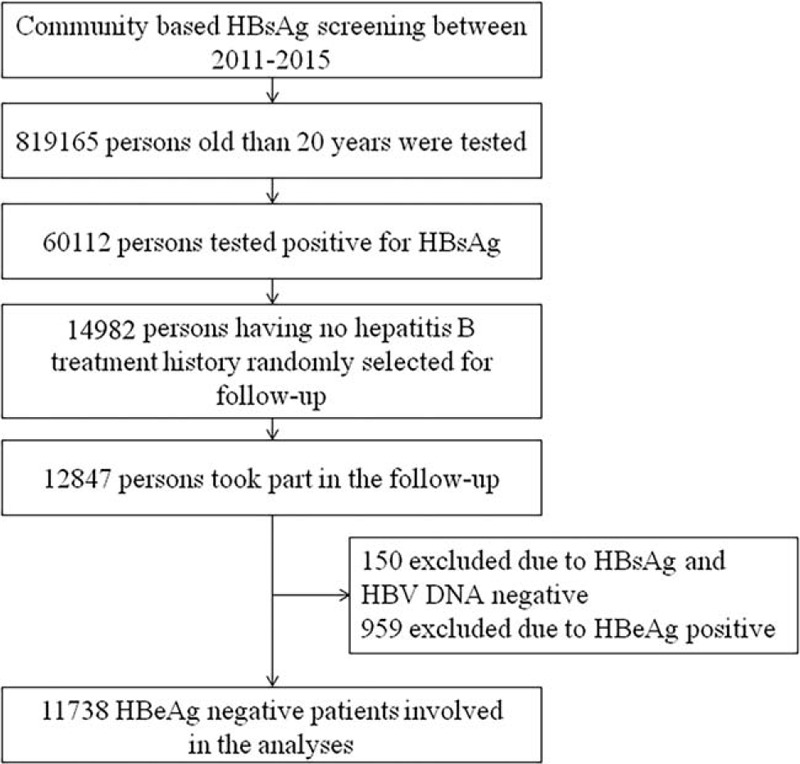
Process of participants enrollment and exclusion. HBeAg = hepatitis B e antigen, HBsAg = hepatitis B surface antigen.

Between 2011 and 2015, a large-scaled community-based free HBsAg screening program was carried out in 50 towns in Zhangjiagang, Danyang, and Taixing in Jiangsu province, China. Since the HBsAg-positive rate among people younger than 20 years has been lower than 2.0% in China,^[5]^ the individuals older than 20-year-old in selected regions were advised to have HBsAg test. By the end of 2015, a total of 819,165 people older than 20-year-old had been tested and 60,112 were HBsAg-positive. Due to the limited financial support, we randomly selected one-fourth of the 60,112 HBsAg-positive people (about 15,000 people) for further follow-up by using the whole group sampling method. In brief, each of the communities (villages) where the HBsAg screenings were performed was assigned a number, and we selected one-fourth of these communities (villages) by randomly choosing numbers. Of the 105 communities (villages) randomly selected, all of the screened HBsAg-positive patients in the selected regions were involved in the further follow-up health examination. In total, 14,982 HBsAg-positive people were finally selected. The other HBsAg-positive people were informed of the results and given a brochure containing further health examination advice on HBV. The selected patients participated in the program by contacting with community doctors or community workers via phone calls or home visits. All the patients were informed of the importance of their health examination about hepatitis B and were provided with a free follow-up health examination. Finally, 12,847 patients (85.7% of the selected people) provided informed consent, and received the examination. Between May 2016 and March 2017, all the follow-up work was finished. After HBV infection biomarkers were tested, 150 (1.2%) people were excluded from current HBV infection due to the both negative HBsAg and HBV-DNA. Finally, 12,697 participants were confirmed chronic HBV infection with longer than 6 months positive results of either HBsAg or HBV-DNA. Among the 12,697 chronic hepatitis B infected patients, 959 patients were HBeAg-positive. Therefore, finally 11,738 HBeAg-negative hepatitis B infected patients were included in the analyses (Fig. 1).

### 2.2 Physical examination and serological testing

During the follow-up health examination, the screened HBsAg-positive patients were referred to a hospital in town, and interviewed by a trained physician using a structured questionnaire to collect data on demographic conditions, history of hepatitis, signs of the hepatitis, etc 10 mL fasting blood samples were obtained by venipuncture for quantitative HBV-DNA test (Sansure Biotech Inc low limit of detection 100 IU/mL, upper limits of detection 5 × 10^9^ IU/mL), routine serum biochemical tests (Shanghai Kehua Bio-engineering Co, Ltd) and HBV serological infection markers tests (Abbort chemiluminescence immunoassay) including HBsAg (upper limits of detection 250 IU/mL), hepatitis B surface antibody (anti-HBs) (upper limits of detection 1000 IU/mL), HBeAg, hepatitis anti-HBe and hepatitis B core antibody (anti-HBc). Upper limits of normal (ULN) of ALT and aspartate aminotransferase (AST) levels were defined as 40 U/L.^[11]^ Serum biochemical tests were conducted in the hospitals where the participants received the health examination within 12 h after the blood samples were collected. Another sub package of the sera were stored in −20°C and transferred to the hepatitis laboratory in Jiangsu Provincial Center for Diseases control and Prevention for HBV serological infection markers tests and quantitative HBV-DNA test.

### 2.3 Data analysis

All the investigation, health examination and laboratory testing data were double-entered into EpiData version 3.02 (EpiData Association, Odense, Denmark) and verified for consistency, and different parts of the data were merged based on participants’ ID. Proportions were used to describe the demographic characteristics of the participants. Student *t* test or one-way analysis of variance was calculated to compare the differences among groups divided by demographic characteristics for continuous normal distributing variables. Chi-square (χ^2^) test was used for categorical variables. The associations of age, sex, HBsAg, and HBV-DNA levels with abnormal liver function risks were estimated by computing the odds ratios (ORs) and their 95% confidence intervals (CIs) from both univariate and multivariate logistic regression analyses. All the statistical analyses were performed with SPSS 13.0 software (SPSS Inc, Chicago, IL), and *P* < .05 in a two-sided test was considered statistically significant.

This field research was approved by the Ethical Review Committee of Jiangsu Provincial Center for Diseases Control and Prevention.

## 3 Results

**Figure 2:**
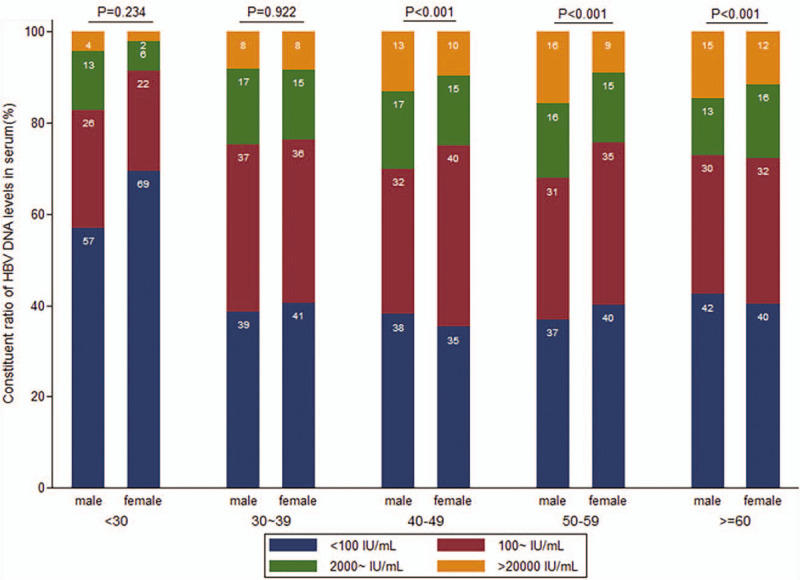
Comparison of HBV-DNA distribution between female and male stratified by age. HBV-DNA = hepatitis B virus deoxyribonucleic acid.

The average age of the patients was 56.1 ± 11.9 years old (male 56.1 ± 12.1, female 56.0 ± 11.8) and 5674 (48.3%) were male. As for HBV-DNA, the mean level was 2.07 log IU/mL and 60% of the patients presented HBV-DNA positive. 27.3% patients had HBV-DNA level higher than 2000 IU/mL, and 11.9% had HBV-DNA level higher than 20,000 IU/mL. HBV-DNA levels tended to increase along with the increasing of age. The male patients had significant higher HBV-DNA levels than female patients; in the age groups older than 40-year-old, the proportion of male patients with higher HBV-DNA levels was significantly larger than that of female patients. The distribution of HBV-DNA levels on selected demographic characteristics was described in Table 1 and Figure 2.

**Table 1:**
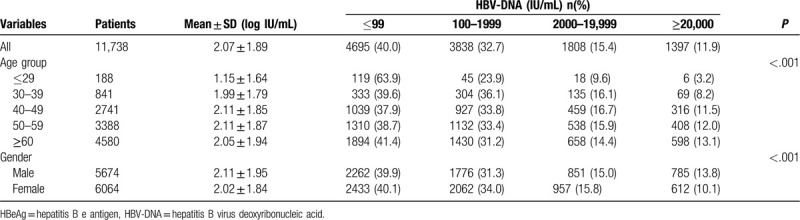
HBV-DNA levels based on demographic and selected variables among the HBeAg negative infection patients.

Among the 11,723 patients having transaminase testing results, 2812 patients (24%) had abnormal ALT or AST. The percentage of abnormal transaminase in male (30.0%) was significantly higher than that in female (18.4%) (*P* < .01), but the occurrences of abnormal transaminase were similar among different age groups. Among patients with HBV-DNA ≥20,000 IU/mL, 55% (769/1397) presented abnormal ALT or AST which was significantly higher than that of patients with HBV-DNA levels below 20,000 IU/mL (19.0–21.7%). Patients with HBsAg higher than 250 IU/mL also had significantly higher percentage of abnormal ALT or AST (28.2%) than that of patients with lower HBsAg levels (19.6–20.5%).

By univariate logistic regression analyses of the risk factors, gender, levels of HBV-DNA, and HBsAg were significantly associated with the risk of abnormal liver function. In multivariate logistic regression analyses, the male patients had 1.87 times of increased risk of having abnormal liver function than female patients. For the HBV infection factors, patients with higher viral load had higher risk of having abnormal liver function. Compared with patients with HBV-DNA below 100 IU/mL, patients with HBV-DNA levels of 100 to 2000 IU/mL had similar risk of having abnormal liver function. The risk rose to 1.16 times higher in patients with HBV-DNA levels of 2000 to 20,000 IU/mL. However, the risk rose sharply to 4.92 times higher in patients with HBV-DNA levels higher than 20,000 IU/mL. As for HBsAg, patients with HBsAg levels below 250 IU/mL had similar risk of having abnormal liver function. Compared with patients with HBsAg levels below 1 IU/mL, patients with HBsAg levels higher than 250 IU/mL had only 1.11 times higher risk of having abnormal liver function. The results were described in Table 2.

**Table 2:**
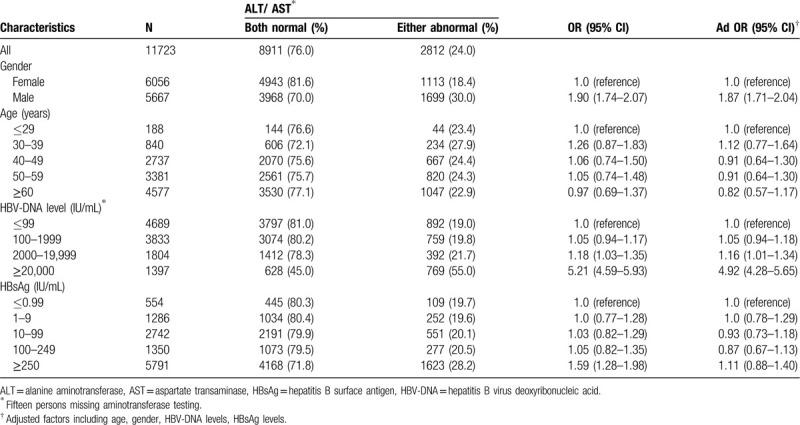
Occurrence of abnormal aminotransferase and the relationship between demographic, virological characteristics and risk of liver damage.

According to 2018 Hepatitis B Guidance of the American Association for the Study of Liver Diseases (AASLD), elevation of ALT ≥2, and the ULN with elevated HBV-DNA above 2000 IU/mL should be used as the standard to decide whether a HBeAg-negative chronic hepatitis B patient should receive antiviral treatment. Based on this standard, 2.1% of 11,723 treatment-naïve HBeAg-negative patients in our study met the indications of antiviral treatment. Among patients with HBV-DNA above 20,000 IU/mL, 15.5% needed treatment, which was significantly higher than that of patients with lower viral load (0.3%). Male patients were more likely to have treatment than female patients (2.9% vs 1.3%, *P* < .001). The percentage of patients needing treatment in different age groups was similar. The results were shown in Table 3.

**Table 3:**
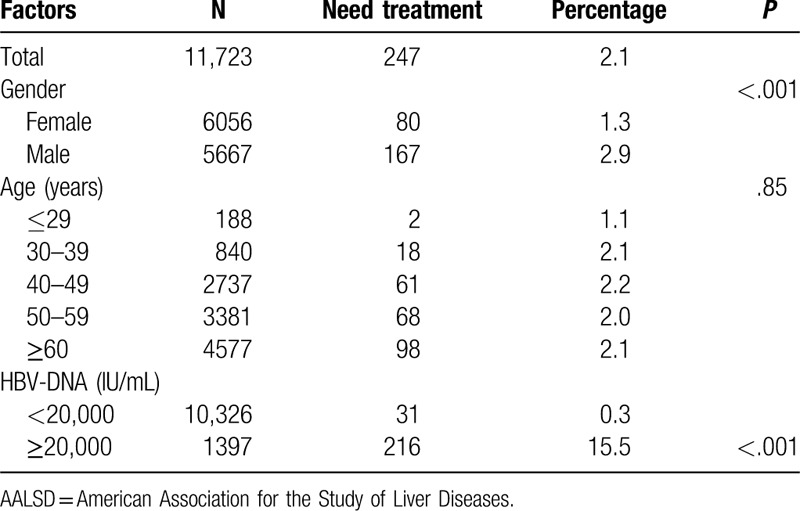
Patients need treatment reference to AASLD 2018 hepatitis B guidance based on demographic, virological characteristics.

## 4 Discussion

In this community-based chronic HBV infection characteristics investigation in adult patients diagnosed from HBsAg screening, it was found that with the average age of 55.4 years, most of the chronic HBV-infected patients were HBeAg-negative (92.4%). Among HBeAg-negative HBV-infected patients, 60.0% were HBV-DNA-positive (≥100 IU/mL), and 27.3% had HBV-DNA levels higher than 2000 IU/mL. As for liver damage, 24% patients presented abnormal transaminase. To the best of our knowledge, the study presented an overall profile of the liver function and viral spectrum of HBeAg-negative chronic HBV infected adults in large-scaled communities in China for the first time.

In our study, the proportion (27.3%) of reactivity of the virus (HBV-DNA ≥2000 IU/mL) in HBeAg-negative chronic HBV-infected patients was higher than that revealed in a similar community-based investigation in Gambia adults (10.7%), even though that research included 3.3% HBeAg-positive patients.^[12]^ The cause of the difference may be the much older age of our participants, which was in accordance with our observation that the virus level increased with age. One reason is that, although the majority of HBeAg-negative patients will remain in an inactive carrier state for a life time, others may develop significant HBV replication over time.^[13,14]^ Another possible reason may be that in older HBeAg-negative patients, some patients may experience late HBeAg seroconversion and have a higher chance of suffering from viral reactivating.^[9,10]^ The geographic distribution of virus genotype may also contribute to the difference. Genotypes E and A contributed the most to the HBV infection in Gambia,^[12]^ while in China, the dominant genotypes are C and B.^[15]^ Among HBeAg-negative patients, the occurrence of pre-core and/or basal core promoter variants was correlated with HBV genotypes.^[16]^ A recent research reported that the HBeAg-negative patients who harbored the pre-core G1896A single mutation or the pre-core G1896A/G1899A double mutation had higher HBV-DNA levels compared with patients without these mutations. In contrast, patients with the BCP A1762T/G1764A mutation showed lower HBV-DNA levels compared with patients without this double mutation.^[17]^ Our research also revealed that the male patients had higher HBV-DNA levels than female patients, especially in patients older than 40-year-old. This is in accordance with the findings from a large cohort case–control study in Taiwan.^[18]^ The gender disparity in HBV replication capability was proved to be related to sex hormones in HBV transgenic animal models, and that androgen-stimulated AR increased the overall HBV transcription.^[19,20]^

Using analysis of risk factors related to abnormal liver function, we found that in univariate analyses, female gender, high level of HBV-DNA (≥2000 IU/mL) and high level of HBsAg (≥ 250 IU/mL) were the risk factors for abnormal transaminase, and in multivariate regression, the risk factors remained unchanged except for HBsAg.

It was found that HBV-infected male have ∼5 to 7 times higher risk of developing HBV-related HCC than HBV-infected female in epidemiological research.^[21]^ The mechanisms of gender disparity of HBV-related liver diseases were proposed to be attributed to sex hormone effects on HBV life cycle and HBV-specific immune responses.^[22]^ Our study also revealed that even the impact of HBV-DNA was eliminated, the male HBeAg-negative patients were more likely to develop abnormal liver function than the female patients, and it could be deduced that more frequent liver inflammation injury in male HBV-infected patients put them in higher risk of developing liver cirrhosis or HCC than female patients.

The correlations between serum HBV-DNA level and hepatic injury were various depending on the patients’ infection statuses. In immune tolerant phase, the patients presented normal ALT levels, high viral loads, and positivity of HBeAg. In inactive phases, the patients were characterized with normal ALT levels, low viremia and negative HBeAg. Up to 10% to 25% of chronic inactive HBV-infected adults may suffer from HBeAg-negative hepatitis flare after HBeAg seroconversion, especially in those who experienced late HBeAg seroconversion.^[9,10]^ Our study indicated that the HBeAg-negative patients presented different viral replication ability and the viral replication ability were associated with the risk of abnormal liver function, but one thing to be noted is that hepatic injury was documented not only in patients with detectable serum HBV-DNA, but also in some patients with undetectable HBV-DNA. It indicated that even the adult HBV-infected patients were in viral inactive phase, the liver function or liver histology should be monitored regularly. In contrast, we also found that some patients with HBV-DNA levels >2000 IU/mL had normal aminotransferase. It is suggested that these infected patients should be followed up more frequently, with ALT determinations every 3 months and HBV-DNA tested every 6 to 12 months for at least 3 years.^[23]^

During the natural history of chronic hepatitis B, serum HBsAg levels declines progressively from the immune-tolerant to the low replicative phase.^[24,25]^ Quantitative serum HBsAg has been used as a biomarker to stratify the risk of disease progression and predict treatment response mainly in patients receiving pegylated interferon (PEG-IFN) therapy.^[26,27]^ The cohort study in Taiwan also indicated that the high baseline level of HBsAg was in the risk of HCC just among HBeAg-negative patients with low HBV load.^[28]^ As for HBeAg-negative infected patients, the clinical spectrum ranges from inactive carrier status to aggressive chronic hepatitis. Several studies reported that the combination of low HBV DNA (<2000 IU/mL) and low HBsAg levels (<1000 IU/mL) could predict inactive carrier status.^[29,30]^ But our study did not find that the patients with higher HBsAg levels had obviously higher risk of having abnormal liver function. One cause was that more than 50% of our patients had HBsAg level higher than the upper limits of detection of 250 IU/mL, and these samples were not diluted for accurate measurement. It could be deduced that, although HBsAg level was relatively lower in HBeAg-negative patients, the upper limits of detection of 250 IU/mL of the HBsAg could not differentiate the infection status accurately and were not suitable for determining HBeAg-negative patients’ clinical status in the follow-up practice.

Another problem revealed was that more than 70% patients in our study were the first diagnosed through HBsAg screening sponsored by NSTMP (unpublished data), which was too late because most of the HBV infections occurred before childhood in China.^[2]^ It was also the problem that some patients missed the treatment intervention. World health organization (WHO) issued its first guideline about HBV and HCV testing to complement the previous issued guideline about prevention, care and treatment of chronic HBV infection.^[31,32]^ Along with the continuous care about chronic HBV infection, these guidelines recommended HBV infection screening in the population with HBsAg >2%, and that confirmed chronic HBV-infected patients should be monitored for further healthcare. So, it is urgent to improve the current situation between high prevalence of chronic HBV infection and lack of HBV screening and further care in China.

The major limitation in our study was that the cross-sectional design and one-time examination might cause misdiagnosis or missed diagnosis of the clinical stages. Another limitation was that the HBsAg quantities of some patients with high titers were not tested with more dilution, which might result in an inaccurate description of the HBsAg quantity distribution and weaken the accuracy of the testing results.

In conclusion, the community-based investigation revealed that in adult HBeAg-negative chronic HBV-infected patients, a high percentage of patients was virological active and the virus activity was related to liver damage. In addition, most of the HBV-infected patients had no regular health examination and some patients missed the vital antiviral treatment. It is necessary and urgent to implement regular active interventions on these chronic HBV-infected patients.

## Acknowledgments

The authors thank the Centers for Disease Control and Prevention in the counties of Zhangjiagang, Danyang, and Taixing for their strong support during this survey. All authors have full access to all of the data in the study and take full responsibility for the integrity of the data and the accuracy of the analysis.

## Author contributions

**Investigation:** Liguo Zhu, Jie Jiang, Ci Song, Hong Peng, Jiao Qian, Yan Zhou, Qungang Wang, Jianfang Xu, Zhijian Wang, Hongjian Liu, Min Fan.

**Methodology:** Xiangjun Zhai.

**Project administration:** Xiangjun Zhai.

**Supervision:** Minghao Zhou, Zhibin Hu, Hongbin Shen, Fengcai Zhu.

**Writing – original draft:** Xiangjun Zhai.
